# Data-Driven Modeling of Cholinergic Modulation of Neural Microcircuits: Bridging Neurons, Synapses and Network Activity

**DOI:** 10.3389/fncir.2018.00077

**Published:** 2018-10-09

**Authors:** Srikanth Ramaswamy, Cristina Colangelo, Henry Markram

**Affiliations:** Blue Brain Project (BBP), École Polytechnique Fédérale de Lausanne (EPFL) Biotech Campus, Geneva, Switzerland

**Keywords:** neuromodulation, acetylcholine, neocortex, microcircuits, cellular excitability, synaptic transmission, network activity

## Abstract

Neuromodulators, such as acetylcholine (ACh), control information processing in neural microcircuits by regulating neuronal and synaptic physiology. Computational models and simulations enable predictions on the potential role of ACh in reconfiguring network activity. As a prelude into investigating how the cellular and synaptic effects of ACh collectively influence emergent network dynamics, we developed a data-driven framework incorporating phenomenological models of the physiology of cholinergic modulation of neocortical cells and synapses. The first-draft models were integrated into a biologically detailed tissue model of neocortical microcircuitry to investigate the effects of levels of ACh on diverse neuron types and synapses, and consequently on emergent network activity. Preliminary simulations from the framework, which was not tuned to reproduce any specific ACh-induced network effects, not only corroborate the long-standing notion that ACh desynchronizes spontaneous network activity, but also predict that a dose-dependent activation of ACh gives rise to a spectrum of neocortical network activity. We show that low levels of ACh, such as during non-rapid eye movement (nREM) sleep, drive microcircuit activity into slow oscillations and network synchrony, whereas high ACh concentrations, such as during wakefulness and REM sleep, govern fast oscillations and network asynchrony. In addition, spontaneous network activity modulated by ACh levels shape spike-time cross-correlations across distinct neuronal populations in strikingly different ways. These effects are likely due to the regulation of neurons and synapses caused by increasing levels of ACh, which enhances cellular excitability and decreases the efficacy of local synaptic transmission. We conclude by discussing future directions to refine the biological accuracy of the framework, which will extend its utility and foster the development of hypotheses to investigate the role of neuromodulators in neural information processing.

## Introduction

The neocortex is densely innervated by cholinergic neurons projecting from the basal forebrain, which release Acetylcholine (ACh; Mesulam et al., 1983; Levey et al., 1987; Gielow and Zaborszky, 2017). Diffuse release of ACh targets neurons and synapses in neocortical microcircuits, and regulates behavioral states, such as attention, wakefulness, learning and memory (Metherate et al., 1992; Hasselmo, 1995, 1999; Lee and Dan, 2012). It is thought that the actions of ACh on the physiology of neurons and synapses plays a key role in switching cortical rhythms that underlie a diversity of behavioral states (McCormick, 1992; Steriade et al., 1993; Juliano and Jacobs, 1995; Xiang et al., 1998; Picciotto et al., 2012; Zagha and McCormick, 2014).

Much of our knowledge on the regulation of neuronal and synaptic physiology by ACh comes from studies in cortical slices that have combined whole-cell somatic recordings and bath-application of ACh agonists, such as carbachol (CCh), to the extracellular recording medium (Wang and McCormick, 1993; Kawaguchi, 1997; Gulledge and Stuart, 2005; Gulledge et al., 2007, 2009; Levy et al., 2008; Eggermann and Feldmeyer, 2009; Brombas et al., 2014; Chen et al., 2015; Poorthuis et al., 2018; Urban-Ciecko et al., 2018). Emerging data suggest that ACh controls the excitability of neocortical neurons, enhances the signal-to-noise ratio of cortical responses, and modifies the threshold for activity-dependent synaptic modifications by activating postsynaptic muscarinic (mAChR) or nicotinic (nAChR) receptors. At the cellular level, it is understood that ACh mostly activates mAChRs to depolarize neurons and initiate action potentials (APs; Krnjević et al., 1971; Kawaguchi, 1997; Gulledge et al., 2009; Eggermann et al., 2014). However, a handful of studies also suggest that ACh transiently activates mAChRs and strongly inhibits the initiation of APs in neocortical pyramidal neurons (Gulledge and Stuart, 2005; Gulledge et al., 2007). At the level of synapses, it is known that ACh reduces the efficacy of excitatory connections in the neocortex. For example, in synaptic connections between thick-tufted layer 5 pyramidal cells (TTPCs), which are marked with pronounced short-term depression, bath-application of 5–10 μM of CCh during presynaptic stimulation, rapidly reduces the rate of depression in a train of postsynaptic potentials (PSPs) without affecting the so-called stationary PSPs (Tsodyks and Markram, 1997; Levy et al., 2008). In contrast, administering a similar amount of CCh on facilitating synaptic connections between TTPCs and Martinotti cells (MCs) increases the strength of successive PSPs (Levy et al., 2008). Although some of the cell-type, and connection-type specific effects of ACh in the neocortex have been experimentally mapped, the vast majority remains unknown.

It is thought that ACh and its interactions with other neuromodulators such as dopamine, noradrenaline and histamine is important in regulating cognitive functions including arousal and attention, sleep-wake cycles, reward, learning and memory (Blandina et al., 2004; Calabresi et al., 2006; Lester et al., 2010; Constantinople and Bruno, 2011). Yet, it has been difficult to develop a unifying view of how ACh controls neuronal and synaptic physiology and impacts neocortical network dynamics. An impediment in this direction is probably due to the fact that ACh differentially controls the activity of neocortical neurons and synapses in complex ways, making it difficult to reconcile its systemic effects (Muñoz and Rudy, 2014). Computational models of neocortical microcircuitry at the cellular and synaptic level of biological detail not only offer an integrative platform to bring together experimental data capturing the specific effects of ACh on dendrites, neurons and synapses, but also make it possible to generate predictions on the actions of ACh at the network level.

**Figure 1:**
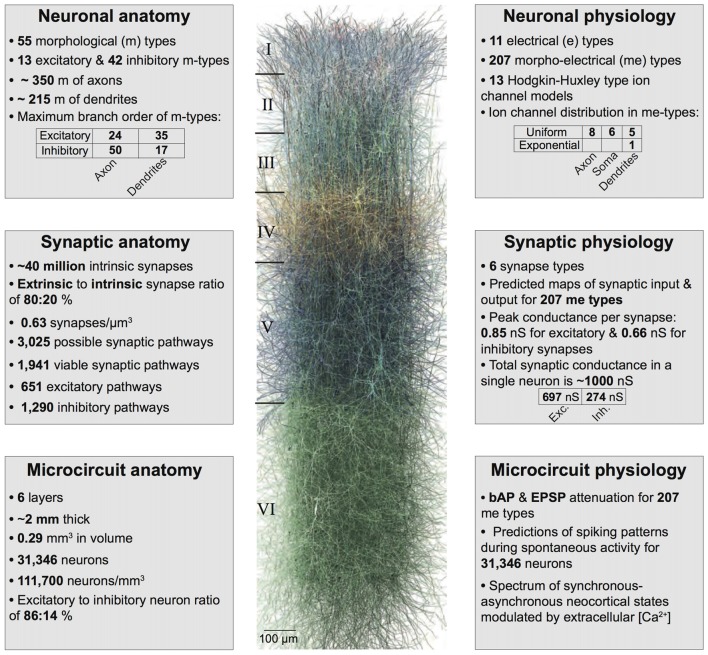
Summary of the biologically detailed tissue model of neocortical microcircuitry. Top left: overview of neuronal anatomy in the reconstruction. Top right: summary of neuronal physiology. Middle left: overview of synaptic anatomy. Middle right: fact and figures on synaptic physiology. Bottom left: summary of microcircuit anatomy. Bottom right: overview of microcircuit physiology.

**Figure 2:**
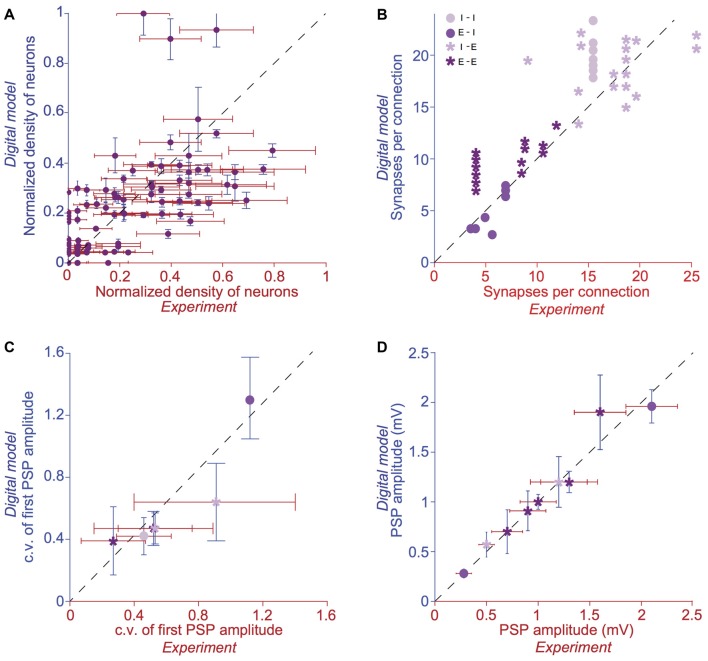
Validation of anatomical and physiological properties in the tissue model of neocortical microcircuitry. **(A)** Normalized neuronal densities. Number of stained neurons per 100 μm bin from layers 1 to 6. Red: *experiment* (counts/bin), blue:* digital model* (counts/bin; mean ± SD, *N* = 100 bins). Dashed line has unit slope. **(B)** Mean number of synapses per connection in excitatory-excitatory (E-E), excitatory-inhibitory (E-I), inhibitory-excitatory (I-E) and inhibitory-inhibitory (I-I) pathways. Red: experiment, blue: digital model. Dashed line has unit slope. **(C)** Mean coefficient of variation (c.v.; defined as standard deviation/mean) of the amplitude of the postsynaptic potential (PSP) for pathways some of the pathways in **(B)**. **(D)** same as in **C**, but for the mean amplitude of the PSP for some of the pathways in **(B)**.

As a way forward, we developed a first-draft, data-driven framework that leverages a recent, rigorously validated digital model of the microcircuitry of juvenile rodent somatosensory cortex (Markram et al., 2015; Ramaswamy et al., 2015; Figure 1) comprising ~31,000 neurons distributed across six layers, 55 layer-specific morphological (m), 11 electrical (e) and 207 morpho-electrical (me) neuron subtypes that are connected through ~40 million synapses and six dynamical synapse (s) types (Figure 2). Next, we augmented the model by integrating the phenomenological cell-type specific effects of ACh neuronal and synaptic physiology from published literature (Kawaguchi, 1997; Tsodyks and Markram, 1997; Gulledge and Stuart, 2005; Gulledge et al., 2007; Levy et al., 2008; Eggermann and Feldmeyer, 2009; Chen et al., 2015). This data-driven approach enabled us to bridge how the local impact of ACh on neurons and synapses are broadcast to the global level and influence the emergence of neocortical network activity. Model parameters were not tuned to replicate any specific ACh-induced network effects. Using this framework, we derive preliminary predictions, which suggest that a dose-dependent change in ACh levels shifts neocortical network state from highly synchronous to asynchronous activity, and distinctly shapes the structure of spike-spike cross-correlations between specific neuronal populations.

## Methods

A digital model of the microcircuitry of juvenile rodent somatosensory cortex was reconstructed as previously described (Markram et al., 2015; Ramaswamy et al., 2015; Reimann et al., 2015). In brief, the reconstruction process comprised the following.

### Microcircuit Dimensions

Thicknesses of individual layers and the diameter of the microcircuit were used to construct a virtual hexagonal prism. A virtual slice was generated from a 1 × 7 mosaic of microcircuits as a cortical sheet with a thickness of 230.9 μm and a width of 2,800 μm.

### Cellular Composition

Measurements of neuronal densities across neocortical layers and fractions of m- and me-types were used to generate the position of individual neurons in the reconstructed microcircuit, constrained by layer-specific proportions of excitatory and inhibitory neurons. Each neuron was assigned the optimal morphology for its location in the microcircuit.

### Digital Neuron Morphologies

Neuronal morphologies were obtained from digital 3D reconstructions of biocytin-stained neurons after whole-cell patch-clamp recordings in 300 μm-thick, sagittal neocortical slices from juvenile rat hind-limb somatosensory cortex. Severed neurites of morphologies due to the slicing procedure were algorithmically regrown (Anwar et al., 2009). Neurites were digitally unraveled to compensate for shrinkage. Neuronal morphologies were then cloned to obtain a sufficient representation of all m-types.

### Electrical Neuron Models

Conductance based, multi-compartmental electrical models of neurons were produced using up to 13 active ion channel mechanisms and a model of intracellular Ca^2+^ dynamics. Axon initial segments (AIS), somata, basal and apical dendrites were modeled as separate, but interconnected compartments. Pyramidal neurons contained two dendritic regions, whereas interneurons contained only one dendritic region. Each region received a separate set of ion channels (see NMC portal^1^; Ramaswamy et al., 2015). With respect to the axon, only the AIS was simulated due to technical limitations in simulating complete axons of all 31,000 neuron models. Each AIS was represented by two fixed length sections, each with a length of 30 μm; diameters were obtained from the reconstructed morphology used for model fitting. APs detected in the AIS were propagated to the synaptic contacts with a delay corresponding to the axonal delay required to propagate to each synapse, assuming an axonal velocity of 300 μm/ms. As previously described, electrical models were fitted using a feature-based multi-objective optimization method.

### Synaptic Anatomy

The number and location of synaptic contacts were derived using an algorithm, described previously (Reimann et al., 2015). The algorithm removes axo-dendritic appositions that do not obey the multi-synapse and plasticity reserve rules and ensures compatibility with biological bouton densities.

### Synaptic Physiology

Excitatory synaptic transmission was modeled using both AMPA and NMDA receptor kinetics. Inhibitory synaptic transmission was modeled with a combination of GABA_A_ and GABA_B_ receptor kinetics. Stochastic synaptic transmission was implemented as a two-state Markov model of neurotransmitter release, a stochastic implementation of the Tsodyks-Markram dynamic synapse model. Biological parameter ranges for the three model parameters—neurotransmitter release probability, recovery from depression and facilitation—were obtained from experimental measurement for synaptic connections between specific m- and me-types or between larger categories of pre and postsynaptic neurons.

### Microcircuit Simulation

The digital model of neocortical microcircuitry was simulated using the NEURON simulation environment, augmented for execution on a supercomputer (Hines and Carnevale, 1997; Hines et al., 2008a,b), along with custom tools to setup and configure microcircuit simulations, and read output results.

We simulated spontaneous background activity by injecting tonic background depolarization to the somata of all neurons, and by modeling miniature PSCs, which were implemented using an independent Poisson process (of rate λ_spont_) at each individual synapse to trigger low release. Spontaneous release rates for inhibitory and excitatory synapses were parameterized to match biological observations (Ling and Benardo, 1999; Simkus and Stricker, 2002). The excitatory spontaneous rate was scaled up per layer to account for missing extrinsic excitatory synapses projecting from subcortical regions, such as the thalamus. The resulting spontaneous release rates for unitary synapses were low enough (0.01 Hz–0.6 Hz) so as not to significantly depress individual synapses.

### Implementation of Dose-Dependent Effects of ACh on Cellular Excitability

Dose-dependent effects of ACh on cellular excitability was achieved by depolarizing somatic step current injection, which caused an increase in the resting membrane potential and firing frequency. Step currents were expressed in terms of percentage of the minimum step current injection required for each cell to spike at least once (rheobase).

### Implementation of Dose-Dependent Effects of ACh on Synaptic Transmission

Dose-dependent effects of ACh on synaptic physiology was achieved by changing the utilization of synaptic efficacy parameter (U) in the stochastic synapse model. The effect of ACh on excitatory and inhibitory synaptic response amplitudes were simulated by modifying the neurotransmitter release probability for all synaptic contacts underlying m-type specific connections according the extrapolated dose-dependence curve compiled from literature. Due to lack of data for specific synaptic connection-types, we assumed that all excitatory and inhibitory connections showed the same dose-dependent effects to ACh.

### Cross-Correlations

Mean spike-spike cross-correlations were computed as the average of all spike-times measured in 10,000–20,000 randomly sampled pairs of excitatory–excitatory (E-E), excitatory–inhibitory (E-I), inhibitory–excitatory (I-E) and inhibitory–inhibitory (I-I) neurons. Cross-correlograms were computed in Matlab (version 9.1).

## Results

### ACh Modulation of Neuronal Physiology

**Figure 3:**
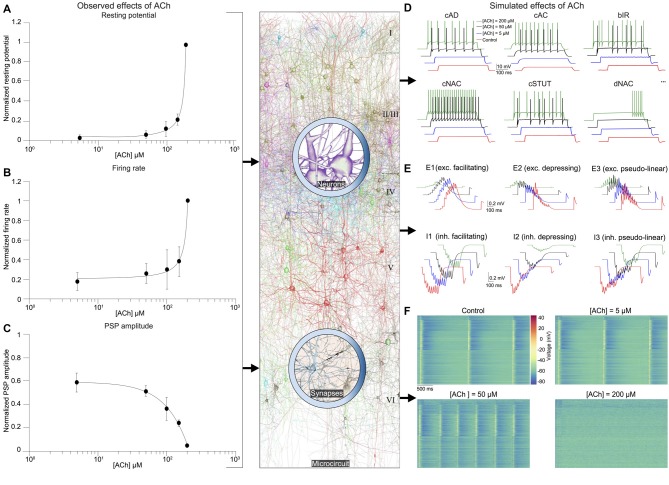
Integrated summary of the cellular, synaptic and microcircuit effects of acetylcholine (ACh) in the tissue model of neocortical microcircuitry. **(A)** Integrated sparse data-sets from published literature on the dose-response effects of ACh on the normalized resting membrane potential of neocortical neurons. Error bars show SD. **(B)** same as in **A**, but for neuronal firing rate. **(C)** same as in **A**, but for the first PSP amplitude. **(D)** Predicted effects of different ACh levels on the resting potential and firing rate of neocortical e-types. Only six e-types are shown. cAD, continuous accommodating (all pyramidal cells); cAC, continuous accommodating (interneurons); bIR, burst irregular, cNAC, continuous non-accommodating; cSTUT, continuous stuttering; dNAC, delayed non-accommodating. **(E)** Predicted effects of different ACh levels on the physiology of all neocortical s-types. **(F)** Prediction of the effect of ACh concentration on network dynamics. Clockwise from left, voltage rasters of 1,000 randomly sampled neurons across layers 1–6 at different ACh concentrations.

Next, we integrated experimental data on the impact of ACh on the resting membrane potential and cellular excitability of neocortical neurons, which enabled us to build a dose-dependent activation profile across a range of ACh concentrations obtained from published literature (Figures 3A,B; see Table 1). We have previously shown that a piece of neocortical tissue, ~0.3 mm^3^ in volume, consists of 55 m-types and 11 e-types, resulting in 207 me-types (for a description of m-, e- and me-types see https://bbp.epfl.ch/nmc-portal/glossary) distributed across six layers (Figure 1). Next, we used validated digital models of 207 me-types that were optimized to reproduce diverse electrophysiological features of excitatory and inhibitory neocortical neurons such as AP amplitudes and widths, mean firing frequency and accommodation index (Ramaswamy et al., 2015; Van Geit et al., 2016). We extended these models by identifying an appropriate level of depolarizing step current injection into the soma, which led to an increase in the resting membrane potential and firing frequency of each me-type to mimic the dose-dependent effects of ACh on cellular excitability (Figure 3D; see “Methods,” section). The amount of injected step current used to simulate cellular excitability at different ACh levels was expressed in terms of percentage of the minimum current injection required for each me-type model to generate at least a single AP (rheobase; see “Methods,” section). In this first-draft implementation of the framework, obtained by augmenting an existing detailed model of neocortical microcircuitry, we began by assuming that all excitatory and inhibitory me-types respond similarly to ACh levels. Excitatory me-types including PCs in all layers and L4 spiny neurons were grouped together. All me-types responded with a change in intrinsic excitability that was predicted to switch from sub-threshold to supra-threshold behavior at an ACh concentration of ~50 μM (Figure 3D; six randomly chosen me-types are shown). The mean AP firing frequency in all me-types increased significantly from 5 Hz to 10 Hz for a four-fold change in ACh from 50 μM to 200 μM (Figure 3D).

**Table 1:** Summary of input data sources on ACh-induced effects on the excitability of neocortical cell-types.

Cell-type	Experimental technique	Physiological effect	References
L23 PC	Bath-application of ~5–100 μM of CCh in Rat/Mouse cortical slices (P13–28)	Prolonged depolarization; increased firing rate	Vidal and Changeux (1993); Levy et al. (2008); Eggermann and Feldmeyer (2009); Chen et al. (2015)
L23 MC	Bath-application of ~10–100 μM of CCh in Rat/Mouse cortical slices (P13–28)	Depolarization; increased firing rate	Chen et al. (2015)
L23 SBC/DBC/BP	Bath-application of ~10–100 μM ACh in Rat/Mouse cortical slices (P18–28)	Depolarization; increased firing rate	Kawaguchi (1997); Chen et al. (2015)
L5 PC	Bath-application of ~100–200 μM ACh in Rat cortical slices (P18–60)	Slow depolarization; Increased firing rate	Gulledge and Stuart (2005); Eggermann and Feldmeyer (2009); Dasari et al. (2017)

### ACh Control of Synaptic Physiology

As the next step, we unified relevant published data, and extrapolated a dose-dependent activation curve of the effects of varying concentrations of ACh on the response amplitude of the first PSP for all neocortical s-types (Figure 3C; see Table 2). It is known that neocortical synapses exhibit at least six distinct forms of excitatory (E) and inhibitory (I) short-term plasticity that are used to distinguish synaptic connections into facilitating (E1 and I1), depressing (E2 and I2), and pseudo-linear (E3 and I3) dynamic s-types (Reyes et al., 1998; Gupta et al., 2000; Feldmeyer et al., 2004; Thomson and Lamy, 2007). We have previously shown that 55 m-types establish around 1,941 morphology-specific synaptic connection types, whose dynamics are governed by one of the six s-types dictated by the pre-post combination of m-types (Markram et al., 2015; Ramaswamy et al., 2015; Reimann et al., 2015). We augmented this model to include the effects of ACh modulation of the first PSP amplitude of s-types and derived predictions on how their short-term facilitating, depressing and pseudo-linear dynamics are controlled by ACh (Figure 3E). It is known that ACh powerfully modulates the PSP amplitude of synaptic connections between excitatory neocortical m-types, very likely by modifying the probability of glutamate release (Levy et al., 2008; Eggermann and Feldmeyer, 2009). However, it remains unclear if ACh controls inhibitory synaptic transmission in the neocortex by modulating GABA release in similar ways to glutamate (Kruglikov and Rudy, 2008; Yamamoto et al., 2010). Therefore, in this first-draft implementation, we assumed that ACh regulates the physiology of both excitatory and inhibitory synaptic connections in comparable ways (Figure 3C; see Table 2).

**Table 2:** Summary of input data sources on ACh-mediated effects on the physiology of neocortical synaptic connections.

Connection-type	Short-term dynamics	Experimental technique	Physiological effect	Reference
L23 PC → L23 BC	E2 Excitatory, depressing	Bath-application of ~5 μM of CCh in Rat S1 slices (P11–26)	Reduction of first PSP amplitude to ~60% of control	Levy et al. (2008)
L23 PC → L23 PC	E2 Excitatory, depressing	Bath-application of ~10 μM oxotremorine or muscarine in Rat A1 slices (P21–28)	Decreases first EPSC amplitude to ~53% of control	Atzori et al. (2005)
L4 excitatory → L4 excitatory	E2 Excitatory, depressing	Bath-application of ~100 μM ACh in Rat S1 slices (P18–24)	Diminishes first PSP amplitude to ~40% of control	Eggermann and Feldmeyer (2009)
L5 PC → L5 PC	E2 Excitatory, depressing	Bath-application of ~100–150 μM ACh in Rat S1 slices (P13–15)	Reduction of first PSP amplitude to ~25% of control	Tsodyks and Markram (1997)
L5 PC → L5 PC	E2 Excitatory, depressing	Bath-application of ~200 μM ACh in Rat S1 slices (P13–15)	Decreases first PSP amplitude to ~5% of control	Tsodyks and Markram (1997)

**Figure 4:**
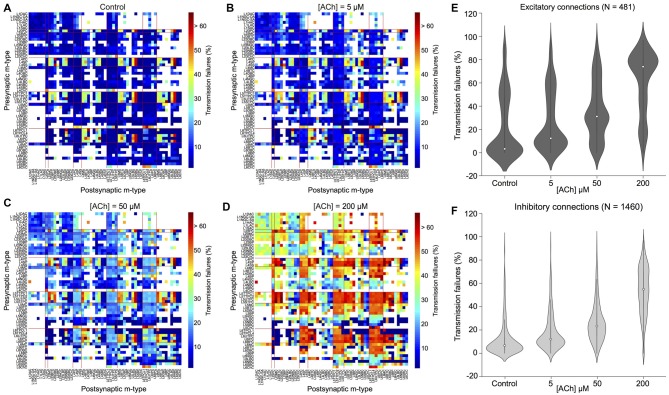
ACh modulates synaptic transmission failures and reorganizes network connectivity. **(A)** A matrix representation of the average synaptic transmission failures for 1,941 connections formed between the 55 m-types (presynaptic on x-axis; postsynaptic on y-axis) in the control condition. Red lines separate excitatory and inhibitory m-types. Black circle shows the L23PC to L23MC connection. **(B)** Same as in **A**, but for [ACh] = 5 μM. **(C)** Same as in **A**, but for [ACh] = 50 μM. **(D)** Same as in **A**, but for [ACh] = 200 μM. **(E)** Violin plot showing the complete probability density distribution of synaptic transmission failures for all excitatory connections (*N* = 481) in the control condition and across different ACh levels. White circle inside the violin plot shows the median of the distribution. Black line shows the interquartile range. **(F)** Same as in **E**, but for all inhibitory connections (*N* = 1,460).

In order to simulate the change in PSP amplitude as a function of ACh concentration, we modified the neurotransmitter release probability for all synaptic contacts underlying m-type specific connections according the extrapolated dose-dependence curve compiled from literature (Figure 3C). We found that ACh exerted highly diverse effects on the PSP amplitude for the six s-types (Figure 3E). The impact of ACh concentrations (5–200 μM) on the first PSP amplitude evoked by injecting a train of nine APs at 30 Hz into the presynaptic soma was superficial compared to control for both E1 (between a L23 PC and a MC) and I1 (between a L23 small basket cell (SBC) and a PC) s-types (Figure 3E, top left; maximum responses are normalized to control). However, the very pronounced facilitation typically observed for the E1 s-type was strongly suppressed at higher (200 μM), rather than lower concentrations (5–100 μM) of ACh (Figure 3E, top left). We found that the amplitude of the first PSP and the subsequent facilitating dynamics for the I1 s-type was not substantially modulated by ACh, despite a four-fold increase in concentration (Figure 3E, bottom left; from 5 μM to 200 μM). The physiology of both E2 (between L5 two thick-tufted pyramidal cells; Figure 4E, top center) and I2 (between a L5 MC and a TTPC; Figure 3E, bottom center) s-types was crucially impacted by different ACh levels (5–200 μM). On average, the first PSP amplitude for both E2 and I2 s-types was reduced to about 75%, 50% and 10% of control at ACh concentrations of 5, 50 and 200 μM ACh, respectively (Figure 3E, top and bottom center). Amplitude of subsequent PSPs decreased to 50%–80% of control with markedly diminished rates of depression, but consistent with previous observations, did not critically impact the amplitude of stationary PSPs (Tsodyks and Markram, 1997; Levy et al., 2008; Eggermann and Feldmeyer, 2009). Higher concentrations of ACh at 200 μM almost completely shutoff depressing synaptic transmission (Figure 3E, top and bottom center). For E3 (between two L6 PCs; Figure 4E, top right) and I3 (between a L5 Nest basket cell (NBC) and a TTPC; Figure 3E, bottom right) pseudo-linear s-types ACh concentrations between 5–100 μM did not cause an increase in the amplitude of the first PSP in a train. At ACh concentrations of 5 and 50 μM, the mean amplitude of the first PSP for E3 and I3 s-types was approximately 70% and 85% of control, respectively (Figure 3E, top and bottom right). Whereas, the amplitude of the first PSP at 200 μM ACh was diminished to about 10% and 50% for E3 and I3 s-types, respectively (Figure 3E, top and bottom right). However, despite an exponential increase in ACh levels from 5 μM to 200 μM the modulation of pseudo-linear dynamics for E3 and I3 s-types appeared to be insensitive to ACh. We predict that an increase in ACh concentration, more than an order of magnitude, has a steep modulatory effect on the physiology of E2 and I2 s-types, but only a superficial impact on E1, I1, E3 and I3 s-types. The diversity of the effects of ACh on the dynamics of the six s-types is somewhat surprising because we implemented homogeneous ACh-induced effects on excitatory and inhibitory synaptic connections in this first-draft framework. Although an exhaustive exploration is beyond the scope this study, it is very likely that the predicted differences in ACh-induced effects on synaptic transmission could arise due to the fact that the anatomical and physiological properties for each of the six s-types in the detailed digital model of neocortical microcircuitry, which forms the foundation for the framework presented here, are quite diverse (Markram et al., 2015). For example, in the detailed digital microcircuit model, there is a large variability in the mean number of synapses for each of the six s-types, which ranges from 5 to 20 contacts per connection, the clear-cut innervation patterns by which synaptic contacts are distributed due to distinct axo-dendritic morphologies, and the specific parameter sets used to model synaptic transmission—peak quantal conductances, release probabilities, time constants for recovery from facilitation and depression (Markram et al., 2015; Ramaswamy et al., 2015; Reimann et al., 2015).

Given that ACh levels modulate the first PSP amplitude by modifying the probability of neurotransmitter release, it should also influence the efficacy and reliability of synaptic transmission. We, therefore, took advantage of our framework to investigate how ACh concentration impacts the reliability of transmission for all 1,941 morphology-specific synaptic connections formed by 55 m-types in neocortical microcircuitry. The average transmission failures for all synaptic connections in the control condition without any ACh was 14.3 ± 19.1% (mean ± SD, *N* = 1, 941 connections), 22.6 ± 27.1% for all excitatory connections (*N* = 481) and 11.6 ± 14.7 for all inhibitory connections (*N* = 1,460). Transmission failures for all synaptic connections at simulated ACh levels of 5, 50 and 200 μM increased nearly fourfold in comparison against control to 20.5 ± 19.3%, 29 ± 19.7% and 55.3 ± 22.6%, respectively. Figures 4A–D shows the predicted average transmission failures for all the 1,941 synaptic connections across different simulated levels of ACh. Upon closer examination, we found that the average transmission failures for all excitatory synaptic connections (*N* = 481) at simulated ACh concentrations of 5, 50 and 200 μM changed nearly threefold compared against control to 27.3 ± 25.5%, 36.3 ± 24.7% and 61 ± 26.7%, respectively (Figure 4E). Transmission failures for all inhibitory connections (*N* = 1,460) at simulated ACh levels of 5, 50 and 200 μM changed nearly fourfold in comparison against control to 18.3 ± 16.2%, 26.6 ± 17.5% and 53.5 ± 20.8%, respectively (Figure 4F). Our preliminary predictions could provide insight on how ACh modulates local cell-type specific connectivity maps between pairs of pre-postsynaptic neurons to reorganize network architecture. In the control case, without ACh, failures between most of the 1,941 morphology-specific synaptic connections are low, which results in highly reliable transmission, and therefore, translates to a higher correlation of a presynaptic spike evoking a postsynaptic response. As ACh concentration increases, failures between synaptic connections increase, which shifts the map of reliable transmission in favor of lower correlation of a presynaptic spike inducing a postsynaptic response.

Experimental studies that have attempted to characterize the effects of ACh on enhancing synaptic properties under *in vivo*-like conditions, in particular transmission failures are few and far between. However, a recent study examined ACh-induced effects on pairs of excitatory L23 PCs and inhibitory somatostatin-expressing neurons (putative MCs) in mouse visual cortex, which are predominantly mediated by weak, facilitating synapses (Urban-Ciecko et al., 2018). The study, which undertook paired whole-cell recordings *in vitro* by mimicking *in vivo*-like conditions (high CCh and low Ca^2+^ levels in the extracellular recording medium), and also through endogenous ACh release by optogenetic stimulation *in vivo*, reported that synaptic transmission between these cell-types was marked with high failures, in the order of ~70% on average (Urban-Ciecko et al., 2018). Although, our framework cannot fully mimic *in vivo* states, predictions of average synaptic transmission failures for connections between L23 PCs and MCs at high ACh concentrations (Figure 4D; about 75% at 200 μM) are consistent with experimental findings. Indeed, our results need to be further validated through targeted experiments. However, the predicted non-linear change in transmission failure rates of all synaptic connections as a function of varying ACh levels is rather striking despite an assumption of homogeneous ACh-mediated effects on both excitatory and inhibitory synapses.

### ACh Modulation of Network Activity

It is thought that ACh enhances arousal and vigilance in primary sensory cortices by altering the signal-to-noise ratio of incoming synaptic input (Minces et al., 2017). However, it remains unclear how the differential regulation of neuronal and synaptic physiology by ACh, specifically the modulation of feedforward excitatory and feedback inhibitory transmission, influences the emergence of neocortical network activity. Previous work has shown that failure of synaptic transmission leads to a suppression of firing rate oscillations and network synchrony (Rosenbaum et al., 2014). In the next set of simulations, we investigated the impact of the ACh-induced changes on the physiology of 31,000 neurons and 1,941 morphology-specific synaptic connections in collectively shaping the dynamics of neocortical microcircuitry. We incorporated phenomenological models of ACh control of neuronal and synaptic physiology into a validated digital model reconstruction of neocortical microcircuitry (Markram et al., 2015) and explored how ACh-induced effects on local cells and synapses modulate global network activity. To enable a direct comparison with experimental data obtained from cortical slices on the impact of ACh on cellular excitability and synaptic transmission, we created a virtual slice (with a thickness of ~231 μm; see “Methods,” section) to explore neocortical network activity for a range of ACh concentrations (see “Methods,” section). We simulated spontaneous activity in the virtual slice by applying tonic background depolarization (see “Methods,” section) and found that in the control condition without any extracellular ACh, neocortical network activity exhibited low-frequency (~1.7 Hz), highly synchronous bursts of oscillatory behavior (Figure 3F, top left) akin to previous reports of regular rhythmic activity during slow-wave sleep (Steriade et al., 1993; Sanchez-Vives and McCormick, 2000; Reyes, 2003). ACh concentrations at 5 and 50 μM further diminished the frequency of synchronous oscillatory network activity (Figure 3F, top right and bottom right). At ACh levels of 200 μM, slow oscillatory bursts of synchronous network activity were superseded by irregular asynchronous activity, resembling active waking states (Figure 3F, bottom right). The transition from synchronous to asynchronous neocortical states occurred at ~75 μM. Interestingly, we found that a change in <50 μM of ACh can switch neocortical dynamics from the synchronous to asynchronous state, divulging two distinct network activity regimes. The mechanisms giving rise to this sharp transition of network activity from synchrony to asynchrony are very likely due to alterations brought about by diverse ACh-induced changes in cellular excitability, physiology of 1,941 synaptic connections and transmission failure rates, and highly correlated excitatory synaptic conductance changes across 31,000 neurons that are almost completely abolished by uncorrelated inhibition.

**Figure 5:**
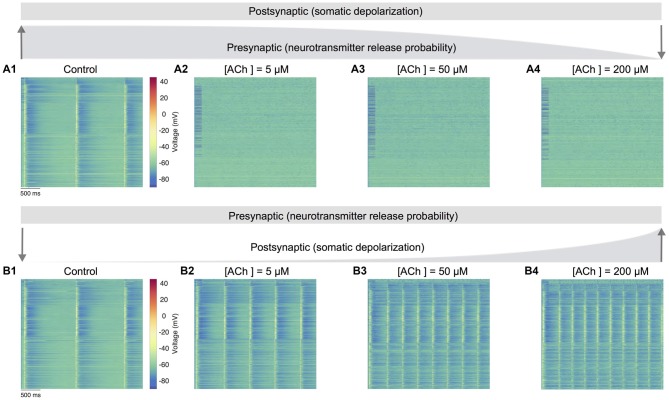
Predicting only the pre- and postsynaptic effects of ACh on network activity. **(A1)** Voltage raster of 1,000 randomly sampled neurons across layers 1–6 with neurotransmitter release probability and somatic depolarization values resembling the control condition. **(A2)** Same as **A1**, but with constant somatic depolarization and neurotransmitter release probability resembling [ACh] = 5 μM. **(A3)** Same as **A1**, but with constant somatic depolarization and neurotransmitter release resembling probability [ACh] = 50 μM. **(A4)** Same as **A1**, but with constant somatic depolarization and neurotransmitter release probability resembling [ACh] = 200 μM. Upward and downward arrows depict the changing gradient of neurotransmitter release probability. **(B1)** Voltage raster of 1,000 randomly sampled neurons across layers 1–6 with neurotransmitter release probability and somatic depolarization values resembling the control condition. **(B2)** Same as in **B1**, but with constant neurotransmitter release probability and somatic depolarization resembling [ACh] = 5 μM. **(B3)** Same as in **B1**, but with constant neurotransmitter release probability and somatic depolarization resembling [ACh] = 50 μM. **(B4)** Same as in **B1**, but with constant neurotransmitter release probability and somatic depolarization resembling [ACh] = 200 μM. Downward and upward arrows depict the changing gradient of somatic depolarization.

Next, we gauged the effects of perturbing only presynaptic (neurotransmitter release probability) or postsynaptic (somatic depolarization) parameters in regulating spontaneous network activity (Figure 5). An extensive parameter sweep of all modeled presynaptic and postsynaptic mechanisms is beyond the scope of this study. We therefore, undertook simple manipulations to explore the impact on network dynamics under two conditions: (1) only the neurotransmitter release probability was gradually changed as before (see “ACh Control of Synaptic Physiology,” section; Figure 3C) to solely simulate the specific presynaptic effects of ACh, but the postsynaptic mechanism achieved through somatic depolarization was fixed at a value matching the control condition in the absence of ACh (see Figures 3A,B); and (2) only the somatic depolarization was gradually varied as above (see “ACh Modulation of Neuronal Physiology,” section; see Figures 3A,B) to exclusively mimic the postsynaptic effects of ACh, but the presynaptic effects attained by changing neurotransmitter release probability was kept constant, again at a value matching the control condition in the absence of ACh (see Figure 3C).

In the first set of simulations, we manipulated only the presynaptic parameter, corresponding to the effects of varying ACh levels exclusively on the neurotransmitter release probability. Expectedly, network activity was highly synchronous at high release probability of all synapses analogous to an absence of ACh in the control case (Figure 5A1). However, continuously altering only the presynaptic parameter through a gradual decrease of neurotransmitter release probability pushed network activity much faster towards asynchrony. Surprisingly, asynchronous network activity remained persistent across changes to presynaptic neurotransmitter release probability resembling low to high ACh levels as before (Figures 5A2–A4). In the next set of simulations, we altered only the postsynaptic parameter reflecting the impact of changing ACh levels specifically on cellular excitability, which was achieved by gradually changing the amount of current required for somatic depolarization as before. In these simulations, the presynaptic parameter was unchanged throughout, and fixed at a high release probability matching the control case. Indeed, it was not surprising that network activity was again synchronous at high release probability and low depolarization levels (Figure 5B1). However, a gradual increase in somatic depolarization levels resulted in network activity becoming more synchronous with an increase in the frequency of oscillatory bursts (Figures 5B2–B4).

Modifying only the presynaptic release probability but keeping postsynaptic somatic depolarization unchanged seems to suggest that the effects of ACh on cellular excitability are essential to gradually, but not abruptly transition network activity from synchrony to asynchrony. An exhaustive analysis of the functional implications of such a sharp transition in network activity is not attempted here. However, from a global standpoint, this sudden shift from synchrony to robust asynchrony could suggest that altered ACh release might lead to sleep disruption, which might result in a failure of memory consolidation (Hasselmo, 1999; Power, 2004; Killgore, 2010). On the other hand, modifying only the postsynaptic depolarization but maintaining a constant presynaptic release probability causes strong, recurrent network activity with a heightened occurrence of oscillatory bursts. This manipulation suggests that the simultaneous effects of an increase in ACh-induced depolarization, which is balanced with a mirroring decrease in neurotransmitter release probability, is crucial to transition network activity from synchrony to asynchrony—for example, in enabling the changeover from non-rapid eye movement (nREM) to REM sleep or waking (Steriade, 2004). Although this warrants further investigation, our preliminary predictions are consistent with previous work showing that a breakdown in the presynaptic effects of ACh could lead to epileptiform-like activity in the neocortex (Benardo, 1991; Schwartzkroin, 1994).

**Figure 6:**
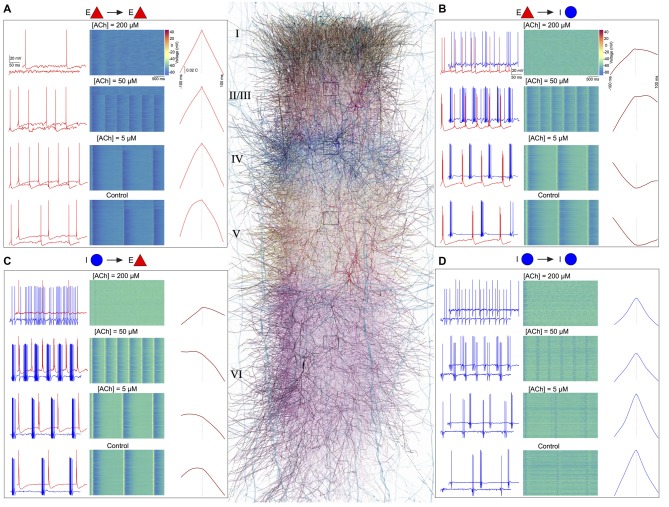
ACh shapes spike-spike cross-correlations. **(A)** Left: spontaneous spiking activity in a randomly chosen pair of E-E (L23 PC-L23 PC) neurons at different ACh levels. Middle: voltage raster for all L23 PCs at different ACh levels. Right: spike-time cross correlations for 10,000–20,000 randomly sampled pairs of L23PCs at different ACh levels. **(B)** same as in **A**, but for E-I neurons (L4 PC-L4 Nest basket cell (NBC)). **(C)** same as in **A**, but for I-E neurons (L5 NBC-L5 thick-tufted layer 5 pyramidal cell (TTPC)). **(D)** Same as in **A**, but for I-I neurons (L6 MC-L6 Martinotti cell (MC)).

Finally, we investigated the effect of ACh concentrations in shaping spike-time cross-correlations for pairs of neurons—E-E (L23 PC-L23 PC; Figure 6A), E-I (L4 PC-L4 NBC; Figure 6B), I-E (L5 NBC-L5 TTPC; Figure 6C), and I-I (L6 MC-L6 MC; Figure 6D). We observed a striking diversity in the average cross-correlation profiles for different pairs of neurons comprising these populations, which was computed as the mean spike-time cross-correlation from 10,000 to 20,000 randomly sampled pairs. At the outset, correlations differed in their temporal profiles (Figures 6A–D). Upon closer examination of these correlation profiles, in particular with the peak lag (delay to peak) and the median lag (delay of the median) revealed that they differed significantly between all examined populations (Figures 6A–D). For example, between pairs of excitatory neurons, the cross-correlations at different ACh concentrations were similar to auto-correlations, with a very small range in peak lag values (Figure 6A) in comparison to the cross-correlations between excitatory and inhibitory neurons (Figure 6B).

Our preliminary results predict that ACh release from subcortical structures, such as the NBM, powerfully modulates neocortical activity giving rise to a spectrum of network activity ranging from one extreme where low ACh levels bring about synchronous activity, to another, where high ACh concentrations lead to asynchrony. Our results are broadly consistent with studies employing optogenetic approaches that associate nREM sleep states with low ACh levels and wakefulness or REM sleep with high ACh concentrations (Lee and Dan, 2012; Chen et al., 2015). We have previously demonstrated that extracellular calcium (Ca^2+^) regulates the emergence of synchronous and asynchronous network activity in the neocortex (Markram et al., 2015). Based on these preliminary predictions, we hypothesize that neuromodulators, such as ACh, provide a complementary functional mechanism in the neocortex, similar to a “push-pull” switch, where the interplay of low ACh and high Ca^2+^ pushes network state towards synchronous activity, whereas high ACh and low Ca^2+^ levels pulls network activity towards asynchrony. We propose that ACh orchestrates neocortical dynamics by generating a spectrum of network activity—where a regime of correlated firing in neurons causes synchronous activity that could modulate functions such as coincidence detection, response selection and binding, and asynchronous activity could promote encoding of new information boosted by heightened attention to incoming sensory input.

## Discussion

This study presents a first-draft implementation of a data-driven framework, which unifies the phenomenological effects of the regulation of local cellular excitability and synaptic physiology by neuromodulators into a data-driven digital model of neocortical microcircuitry to predict their global impact on the emergence of spontaneous network activity, without any parameter tweaking. As a first foray into exploring the utility of this framework, we integrated biological data on how ACh controls the electrical and synaptic properties of cell-types in the rodent neocortex and derived preliminary insights into how a range of ACh levels generated a spectrum of network activity.

Numerous computational models have been proposed to predict cholinergic regulation of network dynamics (Hasselmo, 1993, 1995, 2006; Fellous and Linster, 1998; Tiesinga et al., 2001; Dayan and Yu, 2002; Stiefel et al., 2009; Fink et al., 2011, 2013). However, most of these models have been implemented to specifically replicate distinct behavioral roles of ACh, such as in learning and memory, or by specifically tuning model parameters to match a particular network-level phenomenon. To the best of our knowledge, our data-driven framework, is probably the first bottom-up effort to model cholinergic effects on local cells and synapses and predict emergent global network dynamics, without any parameter tweaking to replicate specific forms of network activity. Our framework, which is an extension of a rigorously validated, detailed biological model of neocortical microcircuitry could, therefore, serve as a substrate to develop hypotheses on the cellular and synaptic mechanisms by which ACh controls network dynamics.

Emerging experimental state-of-the-art suggests that ACh exerts a divergent control of neocortical neurons and synapses. The effects of ACh on the vast majority of neurons and synapses in the neocortex remains unknown. However, the advent of optogenetics to interrogate the cell-type specific effects of ACh combined with a data-driven computational framework, such as the one presented here holds promise in filling knowledge gaps and accelerating our understanding of the complex spatiotemporal actions of ACh in the neocortex.

Although our framework can already provide preliminary insights into ACh regulation of neocortical states by bridging cellular, synaptic and network levels, it is still a first-draft and lacks numerous biological details on the anatomy and physiology of ACh innervation of neocortical layers and neurons. Indeed, in this first-draft implementation, we assumed that the dose-dependent activation profile of ACh is homogeneous on excitatory and inhibitory cell-types and their synaptic connections, which is a gross generalization. For example, recent work reports that ACh inhibits L4 spiny neurons through muscarnic receptors, as against persistent excitation of L23 and L5 PCs (Eggermann and Feldmeyer, 2009; Dasgupta et al., 2018) and could have contrasting effects on sub-types of PCs located in the same neocortical layer and region (Joshi et al., 2016; Baker et al., 2018). As the next step to refine the biological accuracy and specificity of our framework, we plan to systematically incorporate physiological data on cholinergic varicosities, receptor localization and kinetics of ACh receptors, and specific ACh-induced effects on neuronal and synaptic function of an assortment of neocortical cell-types.

A methodical integration of biological data on neuromodulatory control of neocortical cells and synapses into the unifying framework will enable the identification of the unknowns, reconciliation of disparate datasets, and prediction of their general organizing principles. Additionally, our framework not only allows further investigation on the role of ACh in regulating neocortical dynamics but can also be applicable to hypothesize and predict the function of other major neuromodulators—noradrenaline, dopamine, serotonin and histamine—that influence the emergence of network activity. In conclusion, we propose the framework as a complementary resource to existing experimental and theoretical approaches to advance our understanding of how neuromodulatory systems differentially regulate the activity of a diversity of neurons and synapses and sculpt neocortical network activity.

## Author Contributions

SR and HM designed the research. SR and CC integrated data from literature. SR built the framework, models and simulations, made all figures and wrote the manuscript.

## Conflict of Interest Statement

The authors declare that the research was conducted in the absence of any commercial or financial relationships that could be construed as a potential conflict of interest.

## Footnotes

^1^https://bbp.epfl.ch/nmc-portal

## References

[B1] Anwar H. Riachi I. Hill S. Schurmann F. Markram H. (2009). “An approach to capturing neuron morphological diversity,” in Computational Modeling Methods for Neuroscientists, ed. De Schutter E. (Boca Raton, FL: CRC Press), 211–231.

[B2] Atzori M. Kanold P. O. Pineda J. C. Flores-Hernandez J. Paz R. D. (2005). Dopamine prevents muscarinic-induced decrease of glutamate release in the auditory cortex. Neuroscience 134, 1153–1165. 10.1016/j.neuroscience.2005.05.005 16019151

[B3] Baker A. L. O’Toole R. J. Gulledge A. T. (2018). Preferential cholinergic excitation of corticopontine neurons. J. Physiol. 596, 1659–1679. 10.1113/JP275194 29330867 PMC5924837

[B4] Benardo L. S. (1991). Acetylcholine and norepinephrine mediate slow synaptic potentials in normal and epileptic neocortex. Neurosci. Lett. 126, 137–140. 10.1016/0304-3940(91)90538-5 1922924

[B5] Blandina P. Efoudebe M. Cenni G. Mannaioni P. Passani M. B. (2004). Acetylcholine, histamine and cognition: two sides of the same coin. Learn. Mem. 11, 1–8. 10.1101/lm.68004 14747511

[B6] Brombas A. Fletcher L. N. Williams S. R. (2014). Activity-dependent modulation of layer 1 inhibitory neocortical circuits by acetylcholine. J. Neurosci. 34, 1932–1941. 10.1523/jneurosci.4470-13.2014 24478372 PMC6827591

[B7] Calabresi P. Picconi B. Parnetti L. Di Filippo M. (2006). A convergent model for cognitive dysfunctions in Parkinson’s disease: the critical dopamine-acetylcholine synaptic balance. Lancet Neurol. 5, 974–983. 10.1016/S1474-4422(06)70600-7 17052664

[B8] Chen N. Sugihara H. Sur M. (2015). An acetylcholine-activated microcircuit drives temporal dynamics of cortical activity. Nat. Neurosci. 18, 892–902. 10.1038/nn.4002 25915477 PMC4446146

[B9] Constantinople C. M. Bruno R. M. (2011). Effects and mechanisms of wakefulness on local cortical networks. Neuron 69, 1061–1068. 10.1016/j.neuron.2011.02.040 21435553 PMC3069934

[B10] Dasari S. Hill C. Gulledge A. T. (2017). A unifying hypothesis for M1 muscarinic receptor signalling in pyramidal neurons. J. Physiol. 595, 1711–1723. 10.1113/jp273627 27861914 PMC5330881

[B11] Dasgupta R. Seibt F. Beierlein M. (2018). Synaptic release of acetylcholine rapidly suppresses cortical activity by recruiting muscarinic receptors in layer 4. J. Neurosci. 38, 5338–5350. 10.1523/JNEUROSCI.0566-18.2018 29739869 PMC5990982

[B12] Dayan P. Yu A. (2002). “ACh, uncertainty and cortical inference,” in Advances in Neural Information Processing Systems 14, eds Dietterich T. G. Becker S. Ghahramani Z. (Cambridge, MA: MIT Press), 189–196.

[B13] Eggermann E. Feldmeyer D. (2009). Cholinergic filtering in the recurrent excitatory microcircuit of cortical layer 4. Proc. Natl. Acad. Sci. U S A 106, 11753–11758. 10.1073/pnas.0908052106 19564614 PMC2710689

[B14] Eggermann E. Kremer Y. Crochet S. Petersen C. C. H. (2014). Cholinergic signals in mouse barrel cortex during active whisker sensing. Cell Rep. 9, 1654–1660. 10.1016/j.celrep.2014.11.005 25482555

[B15] Feldmeyer D. Egger V. Lübke J. Sakmann B. (2004). Reliable synaptic connections between pairs of excitatory layer 4 neurones within a single ‘barrel’ of developing rat somatosensory cortex. J. Physiol. 521, 169–190. 10.1111/j.1469-7793.1999.00169.x 10562343 PMC2269646

[B16] Fellous J.-M. Linster C. (1998). Computational models of neuromodulation. Neural Comput. 10, 771–805. 10.1162/089976698300017476 9573404

[B17] Fink C. G. Booth V. Zochowski M. (2011). Cellularly-driven differences in network synchronization propensity are differentially modulated by firing frequency. PLoS Comput. Biol. 7:e1002062. 10.1371/journal.pcbi.1002062 21625571 PMC3098201

[B18] Fink C. G. Murphy G. G. Zochowski M. Booth V. (2013). A dynamical role for acetylcholine in synaptic renormalization. PLoS Comput. Biol. 9:e1002939. 10.1371/journal.pcbi.1002939 23516342 PMC3597526

[B19] Gielow M. R. Zaborszky L. (2017). The input-output relationship of the cholinergic basal forebrain. Cell Rep. 18, 1817–1830. 10.1016/j.celrep.2017.01.060 28199851 PMC5725195

[B21] Gulledge A. T. Bucci D. J. Zhang S. S. Matsui M. Yeh H. H. (2009). M1 receptors mediate cholinergic modulation of excitability in neocortical pyramidal neurons. J. Neurosci. 29, 9888–9902. 10.1523/jneurosci.1366-09.2009 19657040 PMC2745329

[B22] Gulledge A. T. Park S. B. Kawaguchi Y. Stuart G. J. (2007). Heterogeneity of phasic cholinergic signaling in neocortical neurons. J. Neurophysiol. 97, 2215–2229. 10.1152/jn.00493.2006 17122323

[B20] Gulledge A. T. Stuart G. J. (2005). Cholinergic inhibition of neocortical pyramidal neurons. J. Neurosci. 25, 10308–10320. 10.1523/jneurosci.2697-05.2005 16267239 PMC6725788

[B23] Gupta A. Wang Y. Markram H. (2000). Organizing principles for a diversity of GABAergic interneurons and synapses in the neocortex. Science 287, 273–278. 10.1126/science.287.5451.273 10634775

[B24] Hasselmo M. E. (1993). Acetylcholine and learning in a cortical associative memory. Neural Comput. 5, 32–44. 10.1162/neco.1993.5.1.32

[B25] Hasselmo M. E. (1995). Neuromodulation and cortical function: modeling the physiological basis of behavior. Behav. Brain Res. 67, 1–27. 10.1016/0166-4328(94)00113-t 7748496

[B26] Hasselmo M. E. (1999). Neuromodulation: acetylcholine and memory consolidation. Trends Cogn. Sci. 3, 351–359. 10.1016/s1364-6613(99)01365-0 10461198

[B27] Hasselmo M. E. (2006). The role of acetylcholine in learning and memory. Curr. Opin. Neurobiol. 16, 710–715. 10.1016/j.conb.2006.09.002 17011181 PMC2659740

[B28] Hines M. L. Carnevale N. T. (1997). The NEURON simulation environment. Neural Comput. 9, 1179–1209. 10.1162/neco.1997.9.6.1179 9248061

[B29] Hines M. L. Eichner H. Schürmann F. (2008a). Neuron splitting in compute-bound parallel network simulations enables runtime scaling with twice as many processors. J. Comput. Neurosci. 25, 203–210. 10.1007/s10827-007-0073-3 18214662 PMC2633940

[B30] Hines M. L. Markram H. Schürmann F. (2008b). Fully implicit parallel simulation of single neurons. J. Comput. Neurosci. 25, 439–448. 10.1007/s10827-008-0087-5 18379867 PMC2760991

[B31] Joshi A. Kalappa B. I. Anderson C. T. Tzounopoulos T. (2016). Cell-specific cholinergic modulation of excitability of layer 5B principal neurons in mouse auditory cortex. J. Neurosci. 36, 8487–8499. 10.1523/jneurosci.0780-16.2016 27511019 PMC4978806

[B32] Juliano S. L. Jacobs S. E. (1995). “The role of acetylcholine in barrel cortex,” in The Barrel Cortex of Rodents, eds Jones I. T. Diamond E. G. (New York, NY: Plenum Press), 411–434.

[B33] Kawaguchi Y. (1997). Selective cholinergic modulation of cortical GABAergic cell subtypes. J. Neurophysiol. 78, 1743–1747. 10.1152/jn.1997.78.3.1743 9310461

[B34] Killgore W. D. S. (2010). “Effects of sleep deprivation on cognition,” in Progress in Brain Research, eds Kerkhof G. A. van Dongen H. P. A. (Amsterdam: Elsevier), 105–129. 10.1016/B978-0-444-53702-7.00007-5 21075236

[B35] Krnjević K. Pumain R. Renaud L. (1971). The mechanism of excitation by acetylcholine in the cerebral cortex. J. Physiol. 215, 247–268. 10.1113/jphysiol.1971.sp009467 5579661 PMC1331876

[B36] Kruglikov I. Rudy B. (2008). Perisomatic GABA release and thalamocortical integration onto neocortical excitatory cells are regulated by neuromodulators. Neuron 58, 911–924. 10.1016/j.neuron.2008.04.024 18579081 PMC2572574

[B37] Lee S.-H. Dan Y. (2012). Neuromodulation of brain states. Neuron 76, 209–222. 10.1016/j.neuron.2012.09.012 23040816 PMC3579548

[B38] Lester D. B. Rogers T. D. Blaha C. D. (2010). Acetylcholine-dopamine interactions in the pathophysiology and treatment of CNS disorders. CNS Neurosci. Ther. 16, 137–162. 10.1111/j.1755-5949.2010.00142.x 20370804 PMC6493877

[B39] Levey A. I. Hallanger A. E. Wainer B. H. (1987). Cholinergic nucleus basalis neurons may influence the cortex via the thalamus. Neurosci. Lett. 74, 7–13. 10.1016/0304-3940(87)90042-5 2436108

[B40] Levy R. B. Reyes A. D. Aoki C. (2008). Cholinergic modulation of local pyramid-interneuron synapses exhibiting divergent short-term dynamics in rat sensory cortex. Brain Res. 1215, 97–104. 10.1016/j.brainres.2008.03.067 18482715 PMC2483424

[B41] Ling D. S. Benardo L. S. (1999). Restrictions on inhibitory circuits contribute to limited recruitment of fast inhibition in rat neocortical pyramidal cells. J. Neurophysiol. 82, 1793–1807. 10.1152/jn.1999.82.4.1793 10515969

[B42] Markram H. Muller E. Ramaswamy S. Reimann M. W. Abdellah M. Aguado Sanchez C.. (2015). Reconstruction and simulation of neocortical microcircuitry. Cell 163, 456–492. 10.1016/j.cell.2015.09.029 26451489

[B43] McCormick D. A. (1992). Neurotransmitter actions in the thalamus and cerebral cortex. J. Clin. Neurophysiol. 9, 212–223. 10.1097/00004691-199204010-00004 1350591

[B44] Mesulam M.-M. Mufson E. J. Levey A. I. Wainer B. H. (1983). Cholinergic innervation of cortex by the basal forebrain: cytochemistry and cortical connections of the septal area, diagonal band nuclei, nucleus basalis (substantia innominata) and hypothalamus in the rhesus monkey. J. Comp. Neurol. 214, 170–197. 10.1002/cne.902140206 6841683

[B45] Metherate R. Cox C. L. Ashe J. H. (1992). Cellular bases of neocortical activation: modulation of neural oscillations by the nucleus basalis and endogenous acetylcholine. J. Neurosci. 12, 4701–4711. 10.1523/jneurosci.12-12-04701.1992 1361197 PMC6575759

[B46] Minces V. Pinto L. Dan Y. Chiba A. A. (2017). Cholinergic shaping of neural correlations. Proc. Natl. Acad. Sci. U S A 114, 5725–5730. 10.1073/pnas.1621493114 28507133 PMC5465883

[B47] Muñoz W. Rudy B. (2014). Spatiotemporal specificity in cholinergic control of neocortical function. Curr. Opin. Neurobiol. 26, 149–160. 10.1016/j.conb.2014.02.015 24637201 PMC4100208

[B48] Picciotto M. R. Higley M. J. Mineur Y. S. (2012). Acetylcholine as a neuromodulator: cholinergic signaling shapes nervous system function and behavior. Neuron 76, 116–129. 10.1016/j.neuron.2012.08.036 23040810 PMC3466476

[B49] Poorthuis R. B. Muhammad K. Wang M. Verhoog M. B. Junek S. Wrana A.. (2018). Rapid neuromodulation of layer 1 interneurons in human neocortex. Cell Rep. 23, 951–958. 10.1016/j.celrep.2018.03.111 29694902 PMC5946807

[B50] Power A. E. (2004). Slow-wave sleep, acetylcholine and memory consolidation. Proc. Natl. Acad. Sci. U S A 101, 1795–1796. 10.1073/pnas.0400237101 14769926 PMC357005

[B51] Ramaswamy S. Courcol J.-D. Abdellah M. Adaszewski S. Antille N. Arsever S.. (2015). The neocortical microcircuit collaboration portal: a resource for rat somatosensory cortex. Front. Neural Circuits 9:44. 10.3389/fncir.2015.00044 26500503 PMC4597797

[B52] Reimann M. W. King J. G. Muller E. B. Ramaswamy S. Markram H. (2015). An algorithm to predict the connectome of neural microcircuits. Front. Comput. Neurosci. 9:120. 10.3389/fncom.2015.00120 26500529 PMC4597796

[B54] Reyes A. D. (2003). Synchrony-dependent propagation of firing rate in iteratively constructed networks *in vitro*. Nat. Neurosci. 6, 593–599. 10.1038/nn1056 12730700

[B53] Reyes A. Lujan R. Rozov A. Burnashev N. Somogyi P. Sakmann B. (1998). Target-cell-specific facilitation and depression in neocortical circuits. Nat. Neurosci. 1, 279–285. 10.1038/1092 10195160

[B55] Rosenbaum R. Zimnik A. Zheng F. Turner R. S. Alzheimer C. Doiron B.. (2014). Axonal and synaptic failure suppress the transfer of firing rate oscillations, synchrony and information during high frequency deep brain stimulation. Neurobiol. Dis. 62, 86–99. 10.1016/j.nbd.2013.09.006 24051279 PMC3877705

[B56] Sanchez-Vives M. V. McCormick D. A. (2000). Cellular and network mechanisms of rhythmic recurrent activity in neocortex. Nat. Neurosci. 3, 1027–1034. 10.1038/79848 11017176

[B57] Schwartzkroin P. A. (1994). Cellular electrophysiology of human epilepsy. Epilepsy Res. 17, 185–192. 10.1016/0920-1211(94)90049-3 8013442

[B58] Simkus C. R. L. Stricker C. (2002). Properties of mEPSCs recorded in layer II neurones of rat barrel cortex. J. Physiol. 545, 509–520. 10.1113/jphysiol.2002.022095 12456830 PMC2290708

[B59] Steriade M. (2004). Acetylcholine systems and rhythmic activities during the waking—sleep cycle. Prog. Brain Res. 145, 179–196. 10.1016/s0079-6123(03)45013-9 14650916

[B60] Steriade M. Amzica F. Nuñez A. (1993). Cholinergic and noradrenergic modulation of the slow (approximately 0.3 Hz) oscillation in neocortical cells. J. Neurophysiol. 70, 1385–1400. 10.1152/jn.1993.70.4.1385 8283204

[B61] Stiefel K. M. Gutkin B. S. Sejnowski T. J. (2009). The effects of cholinergic neuromodulation on neuronal phase-response curves of modeled cortical neurons. J. Comput. Neurosci. 26, 289–301. 10.1007/s10827-008-0111-9 18784991 PMC2857973

[B62] Thomson A. M. Lamy C. (2007). Functional maps of neocortical local circuitry. Front. Neurosci. 1, 19–42. 10.3389/neuro.01.1.1.002.2007 18982117 PMC2518047

[B63] Tiesinga P. H. E. Fellous J.-M. José J. V. Sejnowski T. J. (2001). Computational model of carbachol-induced delta, theta and gamma oscillations in the hippocampus. Hippocampus 11, 251–274. 10.1002/hipo.1041 11769308

[B64] Tsodyks M. V. Markram H. (1997). The neural code between neocortical pyramidal neurons depends on neurotransmitter release probability. Proc. Natl. Acad. Sci. U S A 94, 719–723. 10.1073/pnas.94.2.719 9012851 PMC19580

[B65] Urban-Ciecko J. Jouhanneau J.-S. Myal S. E. Poulet J. F. A. Barth A. L. (2018). Precisely timed nicotinic activation drives SST inhibition in neocortical circuits. Neuron 97, 611.e5–625.e5. 10.1016/j.neuron.2018.01.037 29420933 PMC6588401

[B66] Van Geit W. Gevaert M. Chindemi G. Rössert C. Courcol J.-D. Muller E. B.. (2016). BluePyOpt: leveraging open source software and cloud infrastructure to optimise model parameters in neuroscience. Front. Neuroinform. 10:17. 10.3389/fninf.2016.00017 27375471 PMC4896051

[B67] Vidal C. Changeux J.-P. (1993). Nicotinic and muscarinic modulations of excitatory synaptic transmission in the rat prefrontal cortex *in vitro*. Neuroscience 56, 23–32. 10.1016/0306-4522(93)90558-w 7901807

[B68] Wang Z. McCormick D. A. (1993). Control of firing mode of corticotectal and corticopontine layer V burst-generating neurons by norepinephrine, acetylcholine and 1S,3R- ACPD. J. Neurosci. 13, 2199–2216. 10.1523/jneurosci.13-05-02199.1993 8386756 PMC6576582

[B69] Xiang Z. Huguenard J. R. Prince D. A. (1998). Cholinergic switching within neocortical inhibitory networks. Science 281, 985–988. 10.1126/science.281.5379.985 9703513

[B70] Yamamoto K. Koyanagi Y. Koshikawa N. Kobayashi M. (2010). Postsynaptic cell type-dependent cholinergic regulation of GABAergic synaptic transmission in rat insular cortex. J. Neurophysiol. 104, 1933–1945. 10.1152/jn.00438.2010 20685921

[B71] Zagha E. McCormick D. A. (2014). Neural control of brain state. Curr. Opin. Neurobiol. 29, 178–186. 10.1016/j.conb.2014.09.010 25310628 PMC4254046

